# B Cell Characteristics at Baseline Predict Vaccination Response in RTX Treated Patients

**DOI:** 10.3389/fimmu.2022.822885

**Published:** 2022-04-19

**Authors:** Ana-Luisa Stefanski, Hector Rincon-Arevalo, Eva Schrezenmeier, Kirsten Karberg, Franziska Szelinski, Jacob Ritter, Yidan Chen, Bernd Jahrsdörfer, Carolin Ludwig, Hubert Schrezenmeier, Andreia C. Lino, Thomas Dörner

**Affiliations:** ^1^ Department of Rheumatology and Clinical Immunology, Charité Universitätsmedizin Berlin, Berlin, Germany; ^2^ Deutsches Rheumaforschungszentrum (DRFZ), Berlin, Germany; ^3^ Department of Nephrology and Medical Intensive Care, Charité Universitätsmedizin Berlin, Berlin, Germany; ^4^ Grupo de Inmunología Celular e Inmunogenética, Facultad de Medicina, Instituto de Investigaciones Médicas, Universidad de Antioquia UdeA, Medellín, Colombia; ^5^ Berlin Institute of Health Charité Universitätsmedizin Berlin, Berlin Institute of Health (BIH) Academy, Berlin, Germany; ^6^ Rheumatology Outpatient Office RheumaPraxis Steglitz Berlin, Berlin, Germany; ^7^ Institute of Transfusion Medicine, Ulm University, Ulm, Germany; ^8^ Institute for Clinical Transfusion Medicine and Immunogenetics, German Red Cross Blood Transfusion Service Baden-Württemberg–Hessen and University Hospital Ulm, Ulm, Germany

**Keywords:** RTX (rituximab), B-cells, vaccination, SARS – CoV – 2, prediction

## Abstract

**Background:**

Vaccination is considered as most efficient strategy in controlling SARS-CoV-2 pandemic spread. Nevertheless, patients with autoimmune inflammatory rheumatic diseases receiving rituximab (RTX) are at increased risk to fail humoral and cellular responses upon vaccination. The ability to predict vaccination responses is essential to guide adequate safety and optimal protection in these patients.

**Methods:**

B- and T- cell data before vaccination were evaluated for characteristics predicting vaccine responses in altogether 15 patients with autoimmune inflammatory rheumatic diseases receiving RTX. Eleven patients with rheumatoid arthritis (RA) on other therapies, 11 kidney transplant recipients (KTR) on regular immunosuppression and 15 healthy controls (HC) served as controls. A multidimensional analysis of B cell subsets *via* UMAP algorithm and a correlation matrix were performed in order to identify predictive markers of response in patients under RTX therapy.

**Results:**

Significant differences regarding absolute B cell counts and specific subset distribution pattern between the groups were identified at baseline. In this context, the majority of B cells from vaccination responders of the RTX group (RTX IgG+) were naïve and transitional B cells, whereas vaccination non-responders (RTX IgG-) carried preferentially plasmablasts and double negative (CD27-IgD-) B cells. Moreover, there was a positive correlation between neutralizing antibodies and B cells expressing HLA-DR and CXCR5 as well as an inverse correlation with CD95 expression and CD21low expression by B cells among vaccination responders.

**Summary:**

Substantial repopulation of the naïve B cell compartment after RTX therapy appeared to be essential for an adequate vaccination response, which seem to require the additional capability of antigen presentation and germinal center formation. Moreover, expression of exhaustion markers represent negative predictors of vaccination responses.

## Introduction

Patients with autoimmune inflammatory rheumatic diseases (AIIRD) are at increased risk for infections, attributed to the underlying autoimmune disease, immunosuppressive therapy and comorbidities (1). Thus, COVID-19, caused by the severe acute respiratory syndrome coronavirus-2 (SARS-CoV-2) requires particular considerations in AIIRD patients by rheumatologists. Rituximab (RTX), a first generation anti-CD20 monoclonal antibody leading to B cell depletion and largely used in rheumatologic diseases, has been found as risk factor for poor COVID-19 associated outcomes regarding hospitalization and death (2, 3). Severe COVID-19 can be prevented by vaccination in healthy individuals (4–6), however, B cell depleting therapy with rituximab has been reported to result in substantially diminished vaccination responses following SARS-CoV-2 vaccination (7–9).

The ability to predict vaccination responses is crucial to ensure safety and optimal protection in this patient group. We have previously described that a minimum level of B cell repopulation (at least 10 cells/µl, 0.4% of lymphocytes accordingly) is necessary for RTX treated patients to develop humoral and adequate T cellular responses upon SARS-CoV-2 vaccination (9). In this study, we described predictive markers of vaccination response by assessing qualitative characteristics of the B cell compartment before vaccination (d0) predicting IgG responses by analyzing B cell subsets and molecular B cell markers at baseline.

## Materials and Methods

### Study Participants

**Figure 1:**
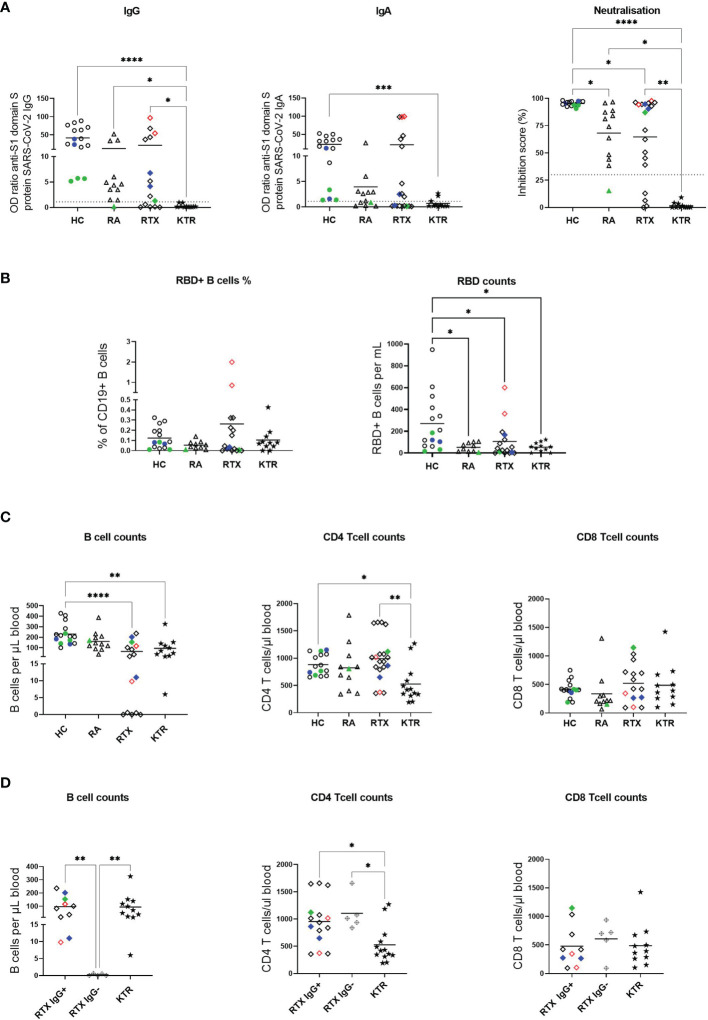
Impaired humoral and cellular anti-SARS-CoV-2 vaccination response in RTX treated patients. **(A)** Humoral immune response against SARS-CoV-2 was assessed by ELISA for spike protein S1 IgG, spike protein S1 IgA and virus neutralization by a blocking ELISA 3 weeks after 2^nd^ SARS-CoV-2 vaccination. Threshold of upper limit of normal is indicated by dotted lines. **(B)** Frequencies and absolute numbers of RBD+ cells among total CD19+ B cells measured 6 ± 3 days after 2^nd^ vaccination. **(C)** Absolute cell counts of CD19+ B cells, CD4+ and CD8+ T cells among the groups at baseline (d0) before vaccination. **(D)** Absolute numbers of CD19+ B cells, CD4+ and CD8+ T cells among the groups at baseline (d0) before vaccination in KTR, RTX IgG+ and RTX IgG- patients. Color code: previously infected individuals are indicated as red quadrats; 2x vaccinated with ChAdOx1 indicated in green; 2x heterologous vaccinated 1x ChAdOx1 followed by 1x BNT162b2, indicated in blue. *p < 0.05, **p < 0.01, ***p < 0.001, ****p < 0.0001.

Outpatient rheumatic patients treated with RTX, who received SARS-CoV-2 vaccination according to federal and Berlin state recommendations between February and May 2021 and participated at our initial study (9), were screened for the availability of baseline data before vaccination (d0). From the previously studied 19 RTX treated patients, we included 13 rheumatoid arthritis (RA) patients [according 2010 ACR Rheumatoid Arthritis Classification Criteria (10)] and 2 ANCA associated vasculitis [AAV patients, defined as (11)] under RTX treatment. Eleven RA patients receiving other therapies (RA group), 11 kidney transplant recipients (KTR) on regular immunosuppression (KTR group) and 15 healthy controls (HC group) served as control groups. All participants gave written informed consent according to the approval of the ethics committee at the Charité University Hospital Berlin (EA2/010/21, EA4/188/20). Peripheral blood samples (EDTA anti-coagulated or serum-tubes, BD Vacutainersystem, BD Diagnostics, Franklin Lakes, NJ, USA) were collected at baseline and 3 weeks after vaccination with either 2x SARS-CoV-2 BNT162b2, 2x ChAdOx1 nCoV-19 or 1x ChAdOx1 nCoV-19 followed by 1x SARS-CoV-2 BNT162b2. Serologic data (**Figure 1A**) and antigen specific (RBD+) B cells (**Figure 1B**) of HC, RA, RTX patients and KTR 3 weeks after 2^nd^ vaccination have been partially previously published (9, 12). Regarding the absolute numbers of CD19+, CD4+ and CD8+ lymphocytes, there was no difference between baseline and after 2nd vaccination (data not shown). Donor information is summarized in **Table 1**.

**Table 1:** Patient characteristics.

	HC N=15	RA N=11	RTX N=15 (13 RA, 2 AAV)	KTR N=11
**Age**				
Median [IQR]	54 [41.5 – 70]	65 [62.5 – 79.5]	59 [57.5 – 64,5]	59 [51.7 – 63]
Under 50	7	2	3	2
Between 50-69	5	4	9	9
> 70	3	5	3	0
**Gender**				
Female	8	8	12	2
Male	7	3	3	9
**Vaccines (n)**				
2x BNT162b2	10	10	12	11
2x ChAdOx1	3	1	1	0
1x ChAdOx1 + 1x BNT162b2	2	0	2	0
**Immunosuppression (n)**				
MTX		6	4	
Leflunomid		1	0	
Sulfasalazin		0	1	
AZA		0	1	
JAKI		4	2	
TNFI		1	0	
Abatacept		1	1	
MMF				11
CNI				10
Prednisolone		2 (max 4mg/d)	6 (max 7.5mg/d)	11 (max. 5mg/d)
**DAS 28**				
Median [IQR]		3.1 [2.4 – 3.5]	2.58 [1,7 – 3.1]	
**Months since last RTX**				
Median [IQR]			8.5 [5.5 – 15]	
**Years on RTX**				
Median [IQR]			3 [1.5 – 6.5]	

IQR, interquartile range; MTX, methotrexate; AZA, azathioprine; JAKI, janus kinase inhibitor; TNFI, tumor necrosis factor alpha inhibitor; MMF, mycophenolate mofetil; CNI, calcineurin inhibitor.

### Enzyme-Linked Immunosorbent Assay

The Euroimmun anti-SARS-CoV-2 assay is a classical enzyme-linked immunosorbent assay (ELISA) for the detection of IgG and IgA to the S1 domain of the SARS-CoV-2 spike (S) protein, and IgG to the SARS-CoV-2 NCP protein. The assay was performed according to the manufacturer´s instructions, as described (12). Briefly, serum samples were diluted at 1:100 in sample buffer and pipetted onto strips of 8 single wells of a 96-well microtiter plate, precoated with recombinant SARS-CoV-2 spike or nucleocapsid proteins. Calibrators, a positive and a negative control were carried out on each plate. After incubation for 60 minutes at 37°C, wells were washed 3 times and the peroxidase-labelled anti-IgG or anti-IgA antibody solution was added, followed by a second incubation step for 30 min. After three additional washing steps, substrate solution was added and the samples incubated for 15 - 30 minutes in the dark. OD values were measured on a POLARstar Omega plate reader (BMG Labtech, Ortenberg, Germany) at 450 nm and at 620 nm. Finally, OD ratios were calculated based on the sample and calibrator OD values. To identify previously SARS-CoV-2 infected individuals we measured antibodies against the nucleocapsid protein (NCP, not a vaccine component) 6 ± 3 days after 2^nd^ vaccination (indicated in red in **Figure 1**).

### Surrogate SARS-CoV-2 Neutralization Test (GenScript)

The assay was performed according to the manufacturer´s instructions, as described (12). This blocking ELISA qualitatively detects anti-SARS-CoV-2 antibodies suppressing the interaction between the receptor binding domain (RBD) of the viral spike glycoprotein (S) and the angiotensin-converting enzyme 2 (ACE2) protein on the surface of cells. After pre-incubation of samples and controls, which allows antibodies in the serum to bind to a horseradish peroxidase (HRP)-conjugated RBD fragment (HRP-RBD), the mixture is added to a capture plate coated with human ACE2 protein. Any unbound HRP-RBD or HRP-RBD bound to non-neutralizing antibodies is captured on the plate. Complexes of neutralizing antibodies and HRP-RBD do not bind on the plate and are removed after three washing steps. Then, TMB is added as a substrate, allowing HRP to catalyze a colour reaction. The colour of the solution changes from blue to yellow after addition of the stop reagent, and can be read by a mictotiter plate reader at 450nm (OD450). The absorbance of the sample is inversely correlated with the amount of SARS-CoV-2 neutralizing antibodies. Positive and negative controls serve as internal controls, the test is considered valid only if the OD450 for each control falls within the respective range (OD450negative control > 1.0, OD450positive control < 0.3). For final interpretation, the inhibition rates were determined using the following formula: Inhibition score (%) = (1 - [OD valuesample/OD valuenegative control] x 100%). Unless stated otherwise, scores < 30% were considered negative, scores ≥ 30% were considered positive.

### Isolation of Peripheral Blood Mononuclear Cells and Staining

PBMCs were prepared by density gradient centrifugation using Ficoll-Paque PLUS (GE Healthcare Bio-Sciences, Chicago, IL, USA). For surface staining 1 x 10^6^ cells were suspended in 50 µl of PBS/0.5% BSA/EDTA and 10 µl Brilliant Buffer (BD Horizon, San Jose, CA, USA). Cells were stained for 15 min on ice and washed afterwards with PBS/0.5% BSA/EDTA (810 xg, 8 min, 4°C).

### Staining of Antigen-Specific B Cells

To identify RBD-specific B cells, recombinant purified RBD (DAGC149, Creative Diagnostics, New York, USA) was labeled with either AF647 or AF488 as reported (9, 12). Double positive cells were considered as antigen-specific. A blocking experiment using unlabeled RBD in 100-fold concentration was used to ensure specificity of detection.

### Flow Cytometry Analysis

All flow cytometric analyses were performed using a BD FACS Fortessa (BD Biosciences, Franklin Lakes, NJ, USA). To ensure comparable mean fluorescence intensities (MFIs) over time of the analyses, Cytometer Setup and Tracking beads (CST beads, BD Biosciences, Franklin Lakes, NJ, USA) and Rainbow Calibration Particles (BD Biosciences, Franklin Lakes, NJ, USA) were used. For flow cytometric analysis, the following fluorochrome-labeled antibodies were used: BUV737 anti-CD11c (BD, clone B-ly6), BUV395 anti-CD14 (BD, clone M5E2), BUV395 anti-CD3 (BD, clone UCHT1), BV786 anti-CD27 (BD, clone L128), BV711 anti-CD19 (BD, clone SJ25C1), BV605 anti-CD24 (BD, clone ML5), BV510 anti-CD10 (BD, clone HI10A), BV421 anti-CXCR5 (BD, clone RF8B2), PE-Cy7 anti-CD95 (ThermoFischer, Waltham, MA, USA clone APO-1/Fas), PE-CF594 anti-IgD (Biolegend, San Diego, CA, USA, clone IA6-2), APC-Cy7 anti-CD38 (Biolegend, clone HIT2), PE-Cy7 anti-IgG (BD, clone G18-145), anti-IgA-Biotin (BD, clone G20-359), BV650 anti-IgM (BD, clone MHM-88), FITC anti-HLA-DR (Biolegend, clone L234), PE anti-CD21 (BD, clone B-ly4), APC anti-CD22 (BD, clone S-HCL-1). The absolute number of B cells, CD4+ and CD8+ T cells was measured with Trucount (BD) and samples were processed according to the manufacturer’s instruction (B cells were defined as CD19+CD45+ CD3-CD14-CD16-CD56- lymphocytes, CD4+ T cells as CD45+CD3+CD4+CD8-CD19- CD14-CD16-CD56- lymphocytes, CD8+ T cells as CD45+CD3+CD8+CD4-CD19- CD14-CD16-CD56- lymphocytes).

### Data Analysis

All samples included in the final analyses had at least 1 × 10^6^ events with a minimum threshold for CD19+ cells of 2,000 events apart from RTX patients: minimal recorded CD19+ events in the RTX group were 13 and 17 events respectively, out of > 1 Mio total recorded events. Flow cytometric data were analyzed by FlowJo software 10.7.1 (TreeStar, Ashland, OR, USA). For UMAP analysis of CD19+ B cells flow cytometry data of all study participants was pre-gated on alive CD19+ B cells, concatenated, down sampled to 350 cells per cohort (total CD19 B cells in the RTX IgG- cohort; DownSmapleV3; FlowJo plugin) and clustered by CD27, IgD, CD38, CD10, CD24. As settings we selected the Euclidean distance function, nearest neighbor value of 15 and a minimum distance of 0.5.

### Statistics

GraphPad Prism Version 5 (GraphPad software, San Diego, CA, USA) was used for statistical analysis. For group comparison Kruskal-Wallis with Dunn´s post-test was used. P-values < 0.05 were considered significant. Correlation matrix was calculated using base R and corrplot package (R Foundation for Statistical Computing) using the Spearman method (n=13 due to limited B cell numbers in 2 RTX patients).

## Results

### Cohorts and Patient Characteristics

For the current vaccination study, we included 15 patients receiving rituximab (13 with rheumatoid arthritis, RA, and 2 with ANCA associated vasculitis, AAV; RTX group), 15 healthy controls (HC group), 11 RA patients on other therapies (RA group) and 11 kidney transplant recipients (KTR group) as additional control groups. The majority of study participants were vaccinated twice with the mRNA vaccine BNT162b2. There were three HC, one RA and one RTX vaccinated twice with the viral vector vaccine ChAdOx1 (indicated in green throughout the figures). Two RTX patients and two HC, respectively received 1x ChAdOx1 followed by a heterologous vaccination with 1x BNT162b2, according to national recommendations (indicated in blue throughout the figures). Demographics and co-medication of all study participants are summarized in **Table 1**. HC were younger than RA patients, but had a comparable age as RTX and KTR patients. The majority of RA and RTX patients were female, while the majority of KTR were male, as characteristic of these patients. At the time of vaccination, RTX patients had received B cell depleting therapy on average for 3 years and median time since the last RTX treatment was 8.5 months. The majority of the KTR patients received triple immunosuppression with mycophenolate mofetil (MMF), a calcineurin-inhibitor (CNI) and low-dose prednisolone.

### Impaired Humoral Response and Induction of RBD+ B Cells Upon SARS-CoV-2 Vaccination in All Patient Groups

Antibody responses to SARS-CoV-2 vaccines were assessed in all individuals, 3 weeks after 2^nd^ vaccination. All HC became positive for anti-S1 IgG and IgA and showed very high (> 90%) SARS-CoV-2 neutralisation. As previously reported (9, 12), KTRs failed to develop IgA and IgG anti-vaccine including neutralizing titres, while in the RA and the RTX group, the titre of neutralizing antibodies were significantly diminished upon vaccination (**Figure 1A**). 10/15 (66.7%) of RTX treated patients compared to 15/15 (100%) of HC, 10/11 (90.9%) of patients in the RA control and 0/11 (0%) of patients from the KTR group mounted anti-spike-IgG SARS-CoV-2 antibodies 3 weeks upon 2nd vaccination. Two RTX patients with unknown prior infection (identified as anti-nucleocapsid protein positive, indicated in red), developed high titers of anti-S1 IgG, IgA and neutralizing antibodies comparable with HC.

Next, we studied SARS-CoV-2 specific B cell responses using flow cytometry to quantify receptor-binding domain (RBD) specific B cells in peripheral blood [as previously described (9, 12), gating strategy shown in **Supplementary Figures 1A, B**]. While percentages of RBD+ B cells were comparable among the groups, there were significantly diminished absolute B cell numbers in all patient groups compared with HC (**Figure 1B**).

### RTX Patients Show Diminished CD19+ B Cell Counts but Normal Range of CD4+/CD8+ T Cell Counts Before Vaccination

To identify predictive factors regarding vaccination response in RTX treated patients, we analyzed cellular data at baseline (d0) before vaccination. First, absolute CD19+, CD4+ and CD8+ cell counts were measured for all groups (**Figure 1C**). While absolute B cell counts were significantly reduced in the RTX and KTR group compared with HC, only KTR showed significantly diminished CD4+ T cell numbers when compared with HC and RTX patients. There were no significant differences regarding CD8+ T cell counts between the groups. A deeper insight into the differences between IgG seroconverted (RTX IgG+), non-seroconverted (RTX IgG-) RTX patients and the KTR group revealed significantly lower B cell counts for the RTX IgG- patients (**Figure 1D**). Furthermore, absolute numbers of CD4+ T cells were significantly diminished in the KTR group compared with RTX IgG+ and RTX IgG- groups.

### Therapy-Related B Cell Subset Distribution Is Characteristic Among the Groups

**Figure 2:**
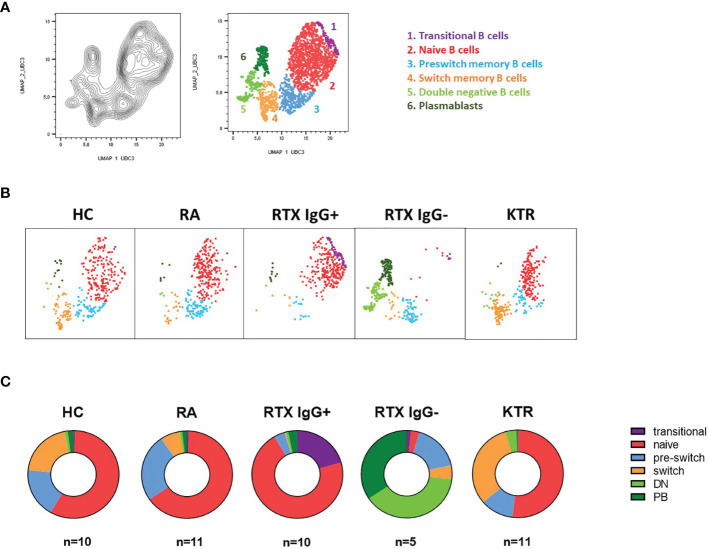
Distinct B cell subsets characterize HC and patient groups. UMAP clustering was performed on a concatenated file of pre-gated CD19+ B cells composed of 350 events in each group. **(A)** Cluster overlay of 1750 B cells of all groups for subset identification. **(B)** Corresponding clusters gated in each donor group. **(C)** Distribution of certain B lineage cell subsets as shown in A showed characteristic differences between the HD, RA, RTX IgG+, RTX IgG- and KTR groups.

Next, we implemented a high-dimensional flow cytometry analysis of circulating B cell populations before vaccination using Uniform Manifold Approximation and Projection (UMAP) dimensionality reduction (**Figure 2**). After down sampling to comparable B cell numbers across all groups, clusters corresponding to distinct subsets of CD19+ B cells were defined as: transitional (CD24+CD38+CD10+), naïve (CD27-IgD+), pre-switch-memory (CD27+IgD+), switched memory (CD27+IgD-) and double negative B cells (CD27-IgD-) as well as plasmablasts (CD27+CD38+, **Figure 2A**, distribution of key markers shown in **Supplementary Figure 2**). Clusters gated in each donor group are shown in **Figures 2B, C**. While the majority of B cells in RTX IgG+ group consisted of naïve and transitional B cells, the predominant subsets in non-responders (RTX IgG-) were plasmablasts and double negative B cells. RA patients on other therapies than RTX and KTR revealed no substantial differences compared to HC, suggesting a specific signal for patients treated with B cell targeted therapy.

### Qualitative B Cell Alterations Before Vaccination Predict Vaccination Response in RTX Treated Patients

**Figure 3:**
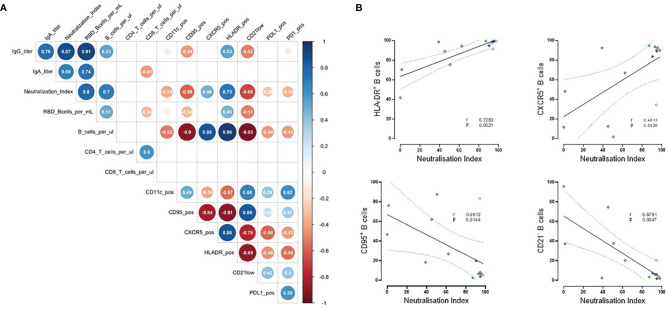
Correlation of humoral vaccine responses, absolute lymphocyte counts and molecule expression in RTX treated patients. **(A)** Spearman´s correlation matrix showing relation between humoral responses, absolute cell counts and expression of CXCR5, HLA-DR, CD95, CD21low, PD-1, PD-L1. Corresponding correlations are represented by red (negative) or blue (positive) circles; size and intensity of color refer to the strength of correlation (RTX n=13 due to limited B cell numbers in 2 RTX patients). Only correlations with p ≤ 0.01 are indicated. Values in circles indicates r value of correlation. **(B)** Significant correlations between neutralizing antibodies and expression of HLA-DR, CXCR5, CD95 and CD21low by B cells from RTX patients.

Next, we screened B cells for the expression of several molecules related to their activation status and functions. Notably, there was a positive correlation between neutralizing antibodies and B cell numbers, HLA-DR and CXCR5 expression on B cells as well as an inverse correlation with CD95 expression and the percentage of CD21low B cells (**Figures 3A, B**). There was no correlation with the expression of PD-1 and PD-L1 on B cells. RBD+ B cell counts correlated with total B cell counts only. There was no significant correlation between CD4+ and CD8+ T cell counts, vaccination response and B cell markers.

## Discussion

Protection through immunization is achieved by an orchestrated immune response between different cellular subsets of innate (APCs) and adaptive immunity, such as B and T cells. Understanding vaccine responsiveness in the context of B cell depleting therapies is essential for preventing infectious diseases in this at-risk patient group. Furthermore, it offers unique insides into B cell biology in general and the function of a B cell impaired immune system in humans.

In the current study, we analyzed the B cell compartment before vaccination in RTX treated patients (13 with RA and 2 with AAV), to identify qualitative predictive markers of an anti-SARS-CoV-2 vaccination response. The RTX patients presented with a wide range of total circulating B cell counts (0.5 - 484/µl blood), providing the opportunity to analyze the B cell compartment at different stages of repopulation. 10/15 patients of the RTX group were able to develop IgG anti-S1-SARS-CoV-2 antibodies upon vaccination, while 5/15 were non-responders. The comparison with the other patient control groups, RA on other therapies (10/11 vaccination responders) and KTR (the majority on triple immunosuppression with MMF/CNI/low-dose-PDN; 11/11 non-responders), addressed the question about the impact of different immunosuppressive therapies.

High-dimensional flow cytometry analysis of B cell subsets before vaccination revealed specific patterns of vaccine prediction between the groups. As known for healthy controls (13), the majority of B cells showed a naïve B-cell phenotype followed by memory compartment (pre-switch, switched memory subsets). Additionally, low numbers of transitional/immature B cells (recent bone marrow migrants), plasma cells and antigen-experienced, double negative B cells were found in peripheral blood.

Other than in HC, the majority of B cells in RTX IgG+ group consisted of naïve and transitional B cells, while the memory compartment counted for less than 10%. More strikingly, the predominant subsets in vaccination non-responding RTX patients were plasmablasts and double negative B cells. Absolute B cell counts and subset distribution suggest that there is still a relevant B cell depletion upon RTX therapy in the RTX IgG- group. Plasmablasts and DN B cells show a lower CD20 expression, and these cells may rather escape CD20 depletion. After rituximab treatment, numerical reconstitution of the B cell compartment is highly variable, but typically begins in 6–9 months. As seen in our cohort, during initial reconstitution, transitional B cells followed by naive B cells predominate in the peripheral blood B cell pool (14). For the immunologic response to neoantigens, like SARS-CoV-2, the diversity within the naive B-cell repertoire appears to be crucial. As evidenced by our study, an adequate vaccination response after RTX therapy required substantial repopulation of naïve B cells with their capacity to differentiate into B lineage memory.

Interestingly, in the KTR cohort, where all patients were vaccination non-responders, we saw significantly reduced total B cell counts, comparable with the RTX IgG+ cohort. While in RTX patients, the repopulation of naïve B cells decides about vaccination response, KTR show also significantly reduced CD4 T cells, which emphasize the more comprehensive effects of the triple immunosuppression and severely limitations in the ability of T cell dependent antibody responses. This analysis reveals the impact of different immunosuppressive therapies (RTX versus MMF/CNI/low-dose-PDN) upon B and T cell compartments critically involved in successful vaccination.

Screening for the expression of several molecules related to B cell activation and functional properties, revealed additional qualitative characteristics, predictive for an adequate vaccination response. The expression level of MHC class II molecules, such as HLA-DR involved in presentation of peptide antigens to T cells, correlated with neutralizing antibodies and B cell counts. Another positive correlation was found for the expression of the chemokine receptor CXCR5 on B cells, which directs B cells into germinal centers and lymphoid tissues. On the other hand, the presence of CD95+ on B cells or CD21low marking exhausted B cells were predictive at baseline for an insufficient vaccination responses in patients who received RTX.

Adding to the known relationship between B cell counts and vaccination response in RTX treated patients, the current study identified qualitative B cell alterations at baseline indicative of their impaired function. The molecules herein identified can serve as predictive biomarkers regarding a successful vaccination in this patient group. Furthermore, we were able to dissect the impact of different immunosuppressive therapies on certain B cell characteristics and their subset distribution.

## Data Availability Statement

The raw data supporting the conclusions of this article will be made available by the authors, without undue reservation.

## Ethics Statement

The studies involving human participants were reviewed and approved by Charité University Hospital Berlin (EA2/010/21, EA4/188/20). The patients/participants provided their written informed consent to participate in this study.

## Author Contributions

The concept of the study was developed by A-LS, AL and TD. Patient’s samples were collected by KK, A-LS, and JR. Data were obtained by A-LS, HR-A, FS, JR, YC, BJ, HS, CL. Data were analyzed by A-LS and H R-A. The theoretical framework was developed by TD, A-LS and AL. The work was supervised by AL, and TD. All authors developed, read, and approved the current manuscript.

## Funding

A-LS is funded by a grant from the German Society of Rheumatology. HR-A holds a scholarship of the COLCIENCIAS scholarship No. 727, 2015. ES received a grant from the Federal Ministry of Education and Research (BMBF) (BCOVIT, 01KI20161). ES is participant in the BIH-Charité Clinician Scientist Program funded by the Charité Universitätsmedizin Berlin and the Berlin Institute of Health. JR is supported by a MD scholarship from the Berlin Institute of Health (BIH). HS received funding from the Ministry for Science, Research and Arts of Baden-Württemberg, Germany (CORE-Project) and the European Commission (HORIZON2020 Project SUPPORT-E, no. 101015756). YC is supported by a state scholarship fund organized by China Scholarship Council.TD received funding by the German Research Foundation (DFG) by projects TRR 130/project 24, Do491/7-5, Do 491/10-1.

## Conflict of Interest

The authors declare that the research was conducted in the absence of any commercial or financial relationships that could be construed as a potential conflict of interest.

## Publisher’s Note

All claims expressed in this article are solely those of the authors and do not necessarily represent those of their affiliated organizations, or those of the publisher, the editors and the reviewers. Any product that may be evaluated in this article, or claim that may be made by its manufacturer, is not guaranteed or endorsed by the publisher.
