# Supplementary material for: B Cell Characteristics at Baseline Predict Vaccination Response in RTX Treated Patients
Source: Front Immunol. 2022 Apr 19;13:822885. doi: 10.3389/fimmu.2022.822885 (PMC9063458; doi:10.3389/fimmu.2022.822885)
Supplement: Supplementary file 1 [file DataSheet_1.pdf]

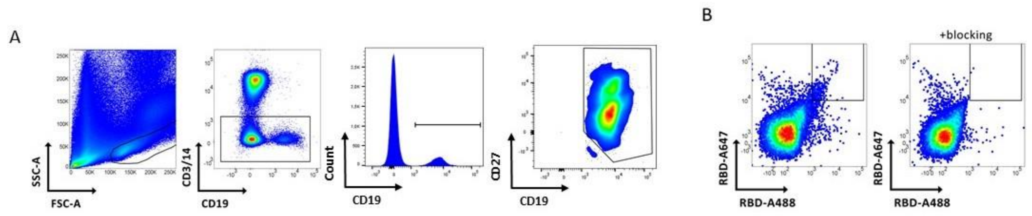

Suppl. Fig1. Representative plots for (A) CD19+ B cells and (B) RBD+ B cells before and after blocking with unlabeled RBD confirming the specificity of their antigen binding.

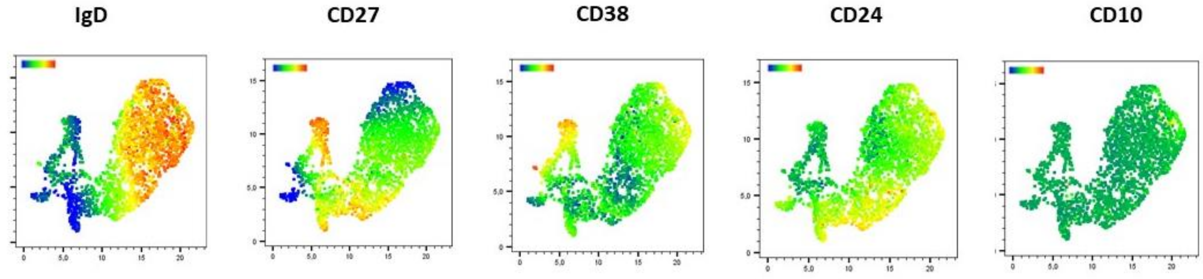

Suppl. Fig2. Distribution of key markers used for defining UMAP cluster (IgD, CD27, CD38, CD24, CD10).
